# Early Prediction of Mortality, Severity, and Length of Stay in the Intensive Care Unit of Sepsis Patients Based on Sepsis 3.0 by Machine Learning Models

**DOI:** 10.3389/fmed.2021.664966

**Published:** 2021-06-28

**Authors:** Longxiang Su, Zheng Xu, Fengxiang Chang, Yingying Ma, Shengjun Liu, Huizhen Jiang, Hao Wang, Dongkai Li, Huan Chen, Xiang Zhou, Na Hong, Weiguo Zhu, Yun Long

**Affiliations:** ^1^Department of Critical Care Medicine, State Key Laboratory of Complex Severe and Rare Diseases, Peking Union Medical College, Peking Union Medical College Hospital, Chinese Academy of Medical Sciences, Beijing, China; ^2^Digital Health China Technologies Co., Ltd., Beijing, China; ^3^Department of Information Center, State Key Laboratory of Complex Severe and Rare Diseases, Peking Union Medical College, Peking Union Medical College Hospital, Chinese Academy of Medical Sciences, Beijing, China; ^4^Department of Primary Care and Family Medicine, State Key Laboratory of Complex Severe and Rare Diseases, Peking Union Medical College, Peking Union Medical College Hospital, Chinese Academy of Medical Sciences, Beijing, China

**Keywords:** sepsis, prediction, machine learning, outcome, sequential (sepsis-related) organ failure assessment

## Abstract

**Background:** Early prediction of the clinical outcome of patients with sepsis is of great significance and can guide treatment and reduce the mortality of patients. However, it is clinically difficult for clinicians.

**Methods:** A total of 2,224 patients with sepsis were involved over a 3-year period (2016–2018) in the intensive care unit (ICU) of Peking Union Medical College Hospital. With all the key medical data from the first 6 h in the ICU, three machine learning models, logistic regression, random forest, and XGBoost, were used to predict mortality, severity (sepsis/septic shock), and length of ICU stay (LOS) (>6 days, ≤ 6 days). Missing data imputation and oversampling were completed on the dataset before introduction into the models.

**Results:** Compared to the mortality and LOS predictions, the severity prediction achieved the best classification results, based on the area under the operating receiver characteristics (AUC), with the random forest classifier (sensitivity = 0.65, specificity = 0.73, F1 score = 0.72, AUC = 0.79). The random forest model also showed the best overall performance (mortality prediction: sensitivity = 0.50, specificity = 0.84, F1 score = 0.66, AUC = 0.74; LOS prediction: sensitivity = 0.79, specificity = 0.66, F1 score = 0.69, AUC = 0.76) among the three models. The predictive ability of the SOFA score itself was inferior to that of the above three models.

**Conclusions:** Using the random forest classifier in the first 6 h of ICU admission can provide a comprehensive early warning of sepsis, which will contribute to the formulation and management of clinical decisions and the allocation and management of resources.

## Introduction

With high morbidity and mortality, sepsis seriously endangers human health and causes a heavy medical burden (1, 2). The understanding of sepsis has evolved from an inflammatory response syndrome caused by infection (sepsis 1.0) to an inflammatory response syndrome with organ dysfunction (sepsis 2.0) to a life-threatening organ disorder caused by the body's uncontrolled response to infection (sepsis 3.0) (3). Employed as the core indicator in sepsis 3.0 diagnosis, the SOFA score was proven to be an accurate and feasible method in the prognosis assessment with its ability to judge the degree of organ failure and assess the severity of patients with sepsis (4, 5). With the establishment of and improvement in critical illness databases and the continuous advancement of machine learning methods, an ever-increasing number of new models are being proposed by researchers. Compared with the SOFA, the Oxford Acute Severity of Illness Score (OASIS) is a scoring system that was constructed by Johnson et al. through machine learning algorithms (6). It contains only 10 variables and no laboratory measure whose diagnostic efficiency is high. Kim et al. (7) also proposed a deep model-based, data-driven early warning score tool, PROMPT, that can predict mortality in critically ill children. With regard to machine learning techniques, Pirracchio et al. proposed that ensemble and neural network models would demonstrate better performance in predicting mortality (8). However, differences exist among the current machine learning models for diagnosis, such as parameter composition, the source population for model construction, and the scope of clinical use. The conclusions obtained by different clinical studies have even been contradictory. This study intends to examine data from the Chinese sepsis patient population under the Chinese medical system and environment using machine learning algorithms to explore a model for predicting the prognosis of sepsis patients, the severity of the disease, and the potential duration of ICU treatment (LOS), which may contribute to understanding sepsis and treating sepsis in the ICU.

## Methods

### Study Design

This study was conducted in the ICU of Peking Union Medical College Hospital. All electronic medical data from patients diagnosed with sepsis based on sepsis 3.0 were retrospectively gathered from 2016 to 2018 and securely stored in the Peking Union Medical College Hospital Intensive Care Medical Information System and Database (PICMISD). The data consisted of demographic information, ICU length of stay (LOS), medications, and vital signs of the respiratory, cardiovascular, hepatic, coagulation, renal, and neurological systems. As one of the commonly used methods for tracking patient status in the ICU and estimating the risk of mortality due to sepsis, a sequential organ failure assessment (SOFA) was introduced as one of the inclusion criteria and a baseline prediction tool. It was computed from the key measurements from multiple-organ systems.

### Patient Cohort

From 2016 to 2018, a total of 11,512 critically ill patients were admitted and treated in the ICU of Peking Union Medical College Hospital. A total of 2,436 patients with sepsis meeting the following criteria were included in the dataset: SOFA score ≥ 2; high possibility of infection (pathogenic microbiology examinations obtained) and usage/update of antibiotics; age ≥ 18 years. After a thorough examination of the dataset, several constraints were added on some variables to ensure the reliability of the medical data: 0 < P(v-a)CO_2_/C(a-v)O_2_ < 5; 0 < P(v-a)CO_2_ < 15; 0 < SO_2_ ≤ 100; 0 < oxygenation index ≤ 1,000; white blood cell ( ×10^8^/L) > 100; oxygen concentration (%) ≥21; and breath rate (bpm) > 0. The number of patients decreased to 2,224 with the extra constraints in place. With reference to the lactic acid values, all patients were labeled as having one of two categories of severity level: sepsis (<2 mmol/L; 1,122 patients) and septic shock (≥2 mmol/L; 1,102 patients). All key measurements of the organs were recorded during the first 6 h after ICU admission. Unlike regular methods of using at least 24 h of measurement in the ICU (9–11), data recorded in the first 6 h can also be sufficiently accurate to assist clinicians in performing early prediction. Informed consent was obtained from all the participants in compliance with the requirements of the Ethics Committee of Peking Union Medical College Hospital.

### Model Development

Regarding the predictor classes, the mortality (survivor, non-survivor) and severity (sepsis, septic shock) predictions depended on the classification model, while patient LOS in the ICU was labeled by dividing patients into two groups: > 6 days and ≤ 6 days. The 6-day cut-off point was derived from the quartile values (first quartile: 3 days, second quartile: 6 days, third quartile: 13 days) from the overall patient distribution. The classification model incorporated the following methods: logistic regression (12), random forest (RF) (13), and XGBoost. To select the most relevant features, the least absolute shrinkage and selection operator (LASSO) was applied. All the features were normalized before being introduced into the classification models. The training and testing datasets were randomly split by 70 and 30% of all patients.

K-nearest neighbor (KNN) imputation (14) was utilized to handle the partial missing data. Each entry of missing data was imputed with the average of its five nearest neighbors. The value k = 5 in the KNN algorithm was chosen because it achieves the best classification results as supported by validation.

As the dataset is enormously biased toward the survivors, a method of over-sampling [specifically, the synthetic minority oversampling technique (SMOTE) (15)] on the minority class was applied in the training dataset for mortality prediction.

The classification models were assessed with the area under the receiver operating characteristic (AUC) curve, sensitivity (also known as recall), specificity, and F1 score. The foundation of these assessment variables comes from the four possible outcomes (TP = true positive, TN = true negative, FP = false positive, FN = false negative) of the binary classifier. Computed by plotting sensitivity as a function of (1-specificity), the area under the ROC curve is widely used as a performance measurement for classification problems at various threshold settings. A higher AUC value indicates a better model for distinguishing between classes. In this study, false positives (e.g., a survivor is predicted as a non-survivor) may be overmedicated, while false negatives (e.g., a non-survivor is predicted as a survivor) may not receive any extra actions for early prevention. Both cases should be avoided here. The F1 score, as the harmonic mean of precision and recall, is a better metric for imbalanced classes. Meanwhile, a five-fold cross validation method was applied for all the models in three classification problems to avoid overfitting during the model training.

$$\begin{array}{lll} Sensitivity & = & \;\frac{TP}{TP+FN}\\ Specificity & = & \;\frac{TN}{TN+FP}\\ F1\;score & = & \;\frac{2^{*}TP}{FP+FN+2^{*}TP} \end{array}$$

### Statistical Analysis

All continuous variables in the clinical data are presented as the mean value ± standard deviation (SD). The distribution of LOS in the ICU was evaluated through quartile values, and then the second quartile value was chosen as the cut-off point for prediction labeling. *T*-tests with a threshold *p* < 0.05 were performed to determine significant differences between subgroups in each prediction problem. Regarding the mortality prediction, the SOFA score, as a baseline prediction tool, was used to generate an ROC curve for comparison with other machine learning models. The sensitivity and specificity of the SOFA score were estimated on the basis of a preset threshold. All statistical analyses were performed in Python 3.6.

## Results

### General Characteristics of Included Patients

A total of 2,224 patients were included in the analysis. Their average of LOS in the ICU was 10.32 ± 11.84 days. The whole group included 1,292 males and 932 females aged 58.96 ± 16.62 years. Approximately 415 (18.7%) patients with sepsis did not survive in the ICU. A summary of the patients' clinical data for each prediction is presented in Tables 1A–C.

**Table 1A:** Subgroups of patients' clinical data for the mortality prediction.

**Variables**	**Mortality**		
	**Survivor (1,809 patients)**	**Non-survivor (415 patients)**	***p*-value**
	**Mean ± SD**	**Mean ± SD**	
Age (years)	58.59 ± 16.82	60.58 ± 15.66	^*^
Perfusion index	1.72 ± 1.74	1.65 ± 1.60	>0.05
P(v-a)CO_2_/C(a-v)O_2_	1.62 ± 0.56	1.63 ± 0.60	>0.05
pCO_2_ (mmHg)	38.02 ± 8.44	38.54 ± 11.53	>0.05
Noradrenaline dosage (μg/kg/min)	0.41 ± 0.74	0.63 ± 1.61	^**^
Adrenaline dosage (μg/kg/min)	0.16 ± 0.08	0.17 ± 0.10	^**^
Invasive blood pressure (mmHg)	92.62 ± 20.50	86.36 ± 18.51	^**^
Central venous pressure (mmHg)	9.25 ± 3.10	9.88 ± 4.00	^**^
P(v-a)CO_2_ (mmHg)	5.49 ± 2.16	5.32 ± 2.28	>0.05
sO_2_ (%)	96.51 ± 4.46	95.77 ± 4.77	^**^
Lactic acid (mmol/l)	2.74 ± 2.73	3.31 ± 3.51	^**^
Invasive systolic blood pressure (mmHg)	139.54 ± 27.81	131.40 ± 28.63	^**^
Invasive diastolic blood pressure (mmHg)	69.88 ± 14.93	65.40 ± 13.73	^**^
Oxygenation index	311.83 ± 143.35	253.27 ± 142.73	^**^
White blood cell (×109/l)	13.83 ± 8.52	14.37 ± 9.38	>0.05
Platelet (×109/l)	176.83 ± 101.13	150.74 ± 105.83	^**^
Total bilirubin (μmol/l)	28.20 ± 47.16	43.21 ± 72.54	^**^
GCS score	9.57 ± 4.48	8.12 ± 4.60	^**^
Creatinine (μmol/L)	121.11 ± 138.15	166.10 ± 156.81	^**^
Oxygen concentration (%)	46.16 ± 16.65	56.33 ± 22.98	^**^
SpO_2_ (%)	97.63 ± 3.58	96.49 ± 4.35	^**^
pO_2_ (mmHg)	108.44 ± 41.27	102.08 ± 47.63	*
Heart rate (bpm)	100.85 ± 21.43	107.21 ± 22.16	^**^
Body temperature (°C)	36.83 ± 1.02	37.21 ± 1.06	^**^
Respiratory rate (bpm)	20.03 ± 6.31	22.79 ± 7.11	^**^
SOFA score	8.82 ± 3.73	11.50 ± 4.27	^**^

** *p < 0.01,*

* *p < 0.05*.

**Table 1B:** Subgroups of patients' clinical data for the severity prediction.

**Variables**	**Severity**		
	**Sepsis (1,104 patients)**	**Septic shock (1,120 patients)**	
	**Mean ± SD**	**Mean ± SD**	***p*-value**
Age (years)	60.47 ± 16.60	57.48 ± 16.52	^**^
Perfusion index	1.93 ± 1.82	1.48 ± 1.56	^**^
P(v-a)CO_2_/C(a-v)O_2_	1.61 ± 0.57	1.63 ± 0.56	>0.05
pCO2 (mmHg)	38.60 ± 10.27	37.64 ± 7.74	^*^
Noradrenaline dosage (μg/kg/min)	0.35 ± 0.24	0.55 ± 1.34	^**^
Adrenaline dosage (μg/kg/min)	0.16 ± 0.03	0.16 ± 0.11	>0.05
Invasive blood pressure (mmHg)	91.59 ± 19.89	91.31 ± 20.68	>0.05
Central venous pressure (mmHg)	9.25 ± 3.03	9.48 ± 3.53	>0.05
P(v-a)CO_2_ (mmHg)	5.34 ± 2.10	5.58 ± 2.26	^*^
sO_2_ (%)	96.62 ± 3.47	96.12 ± 5.36	^*^
Lactic acid (mmol/l)	1.19 ± 0.40	4.49 ± 3.34	^**^
Invasive systolic blood pressure (mmHg)	140.04 ± 28.43	136.02 ± 27.71	^**^
Invasive diastolic blood pressure (mmHg)	68.29 ± 15.00	69.77 ± 14.60	^*^
Oxygenation index	298.95 ± 145.17	302.74 ± 144.91	>0.05
White blood cell (×109/l)	13.40 ± 7.34	14.46 ± 9.81	^**^
Platelet (×109/l)	182.86 ± 107.90	161.18 ± 95.74	^**^
Total bilirubin (μmol/L)	30.52 ± 52.00	31.59 ± 54.29	>0.05
GCS score	9.90 ± 4.41	8.70 ± 4.59	^**^
Creatinine (μmol/L)	128.75 ± 139.59	130.32 ± 146.10	>0.05
Oxygen concentration (%)	47.74 ± 17.99	48.39 ± 18.86	>0.05
SpO_2_ (%)	97.35 ± 3.77	97.48 ± 3.77	>0.05
pO_2_ (mmHg)	103.99 ± 42.34	110.46 ± 42.62	^**^
Heart rate (bpm)	99.61 ± 20.96	104.44 ± 22.16	^**^
Body temperature (°C)	37.03 ± 1.00	36.77 ± 1.07	^**^
Respiratory rate (bpm)	20.56 ± 6.35	20.54 ± 6.76	>0.05
SOFA score	8.66 ± 3.68	9.97 ± 4.14	^**^

** *p < 0.01,*

* *p < 0.05*.

**Table 1C:** Subgroups of patients' clinical data for the LOS prediction.

**Variables**	**LOS**		
	**≤6 days (988 patients)**	**>6 days (1,236 patients)**	
	**Mean ± SD**	**Mean ± SD**	***p*-value**
Age (years)	57.89 ± 16.61	59.82 ± 16.59	^*^
Perfusion index	1.74 ± 1.81	1.67 ± 1.63	>0.05
P(v-a)CO_2_/C(a-v)O_2_	1.64 ± 0.52	1.61 ± 0.61	>0.05
pCO_2_ (mmHg)	37.46 ± 8.06	38.64 ± 9.82	^**^
Noradrenaline dosage (μg/kg/min)	0.53 ± 1.36	0.39 ± 0.45	^**^
Adrenaline dosage (μg/kg/min)	0.17 ± 0.11	0.16 ± 0.05	^**^
Invasive blood pressure (mmHg)	94.49 ± 20.91	89.02 ± 19.44	^**^
Central venous pressure (mmHg)	9.02 ± 3.16	9.64 ± 3.37	^**^
P(v-a)CO_2_ (mmHg)	5.62 ± 2.07	5.32 ± 2.25	^**^
sO_2_ (%)	96.67 ± 4.65	96.13 ± 4.41	^*^
Lactic acid (mmol/l)	2.94 ± 3.11	2.77 ± 2.72	>0.05
Invasive systolic blood pressure (mmHg)	139.41 ± 28.76	136.90 ± 27.59	^*^
Invasive diastolic blood pressure (mmHg)	71.59 ± 15.24	67.00 ± 14.15	^**^
Oxygenation index	337.15 ± 145.09	271.89 ± 138.34	^**^
White blood cell (×109/l)	13.08 ± 8.04	14.62 ± 9.12	^**^
Platelet (×109/l)	174.57 ± 98.89	169.85 ± 105.31	>0.05
Total bilirubin (μmol/l)	33.11 ± 58.50	29.33 ± 48.42	>0.05
GCS score	10.04 ± 4.48	8.71 ± 4.50	^**^
Creatinine (μmol/l)	105.55 ± 119.71	148.69 ± 156.39	^**^
Oxygen concentration (%)	44.31 ± 15.79	51.06 ± 19.80	^**^
SpO_2_ (%)	97.85 ± 3.48	97.07 ± 3.94	^**^
pO_2_ (mmHg)	113.13 ± 43.03	102.55 ± 41.68	^**^
Heart rate (bpm)	99.68 ± 22.26	103.93 ± 21.07	^**^
Body temperature (°C)	36.68 ± 1.00	37.08 ± 1.04	^**^
Respiratory rate (bpm)	19.41 ± 5.96	21.46 ± 6.86	^**^
SOFA score	8.37 ± 3.89	10.08 ± 3.88	^**^

** *p < 0.01,*

* *p < 0.05*

### Mortality Prediction

In the dataset, the number of non-survivors (415 patients) was approximately a quarter of the number of survivors (1,809 patients). The non-survivor group was slightly older than the survivor group. Among the 25 variables in Table 1A, only five variables, including perfusion index, P(v-a)CO_2_/C(a-v)O_2_, pCO_2_, P(v-a)CO_2_, and white blood cell count, showed no significant difference between the two groups, while the remaining variables did. With regular statistical methods, the SOFA score was used to produce ROC curves individually instead of being included as a feature in the model. It is reasonable that the average SOFA score for the survivor group was significantly lower than that for the non-survivor group.

**Figure 1:**
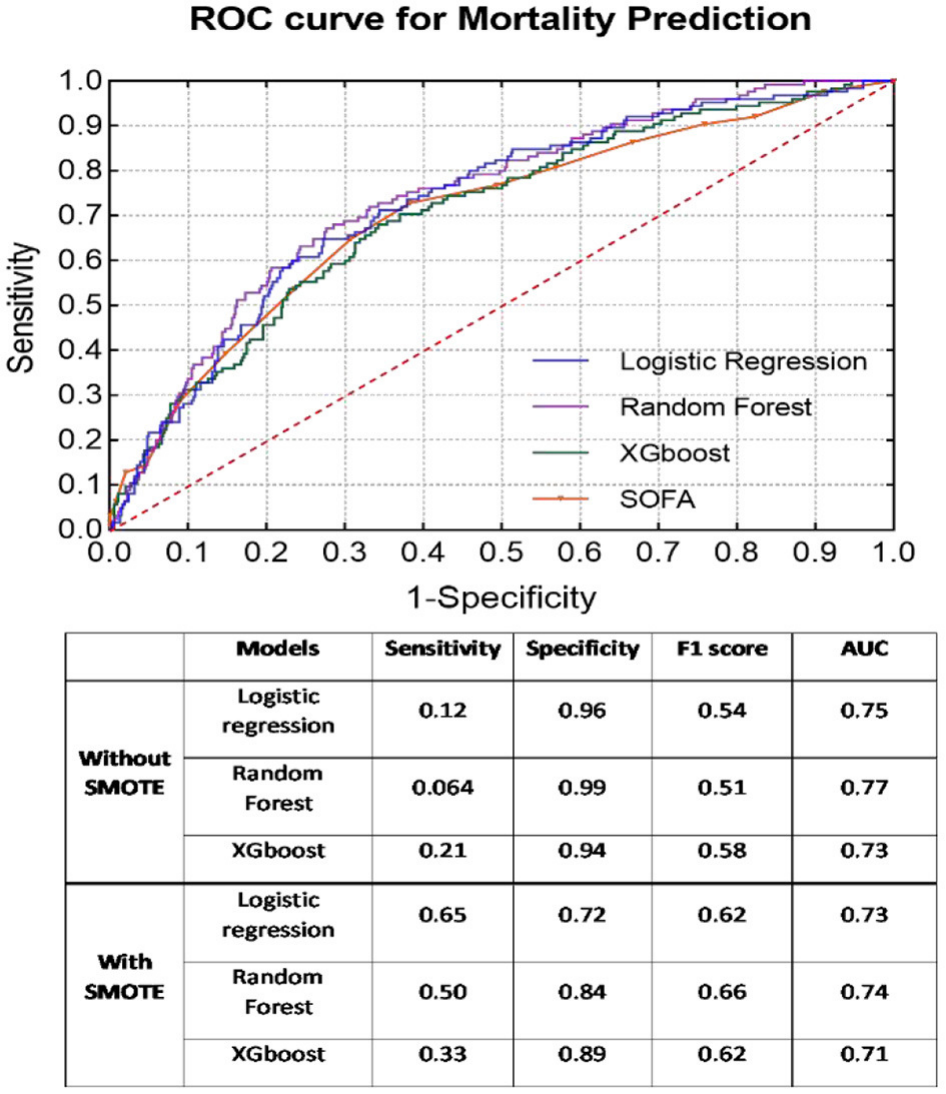
ROC curves of each classifier with SMOTE for mortality prediction. Classification results before and after SMOTE are presented in the embedded table.

As presented Figure 1, the SMOTE method significantly improved the sensitivity rate (without SMOTE: mean sensitivity = 0.13; with SMOTE: mean sensitivity = 0.49) in all models. Nonetheless, specificity, together with AUC, from all three models was considerably reduced after applying the SMOTE method. RF presented the best classification results (without SMOTE: AUC = 0.77; with SMOTE: AUC = 0.74), regardless of the application of the SMOTE method. All machine learning models demonstrated better prediction results than the SOFA score (AUC = 0.70).

### Severity Prediction

**Figure 2:**
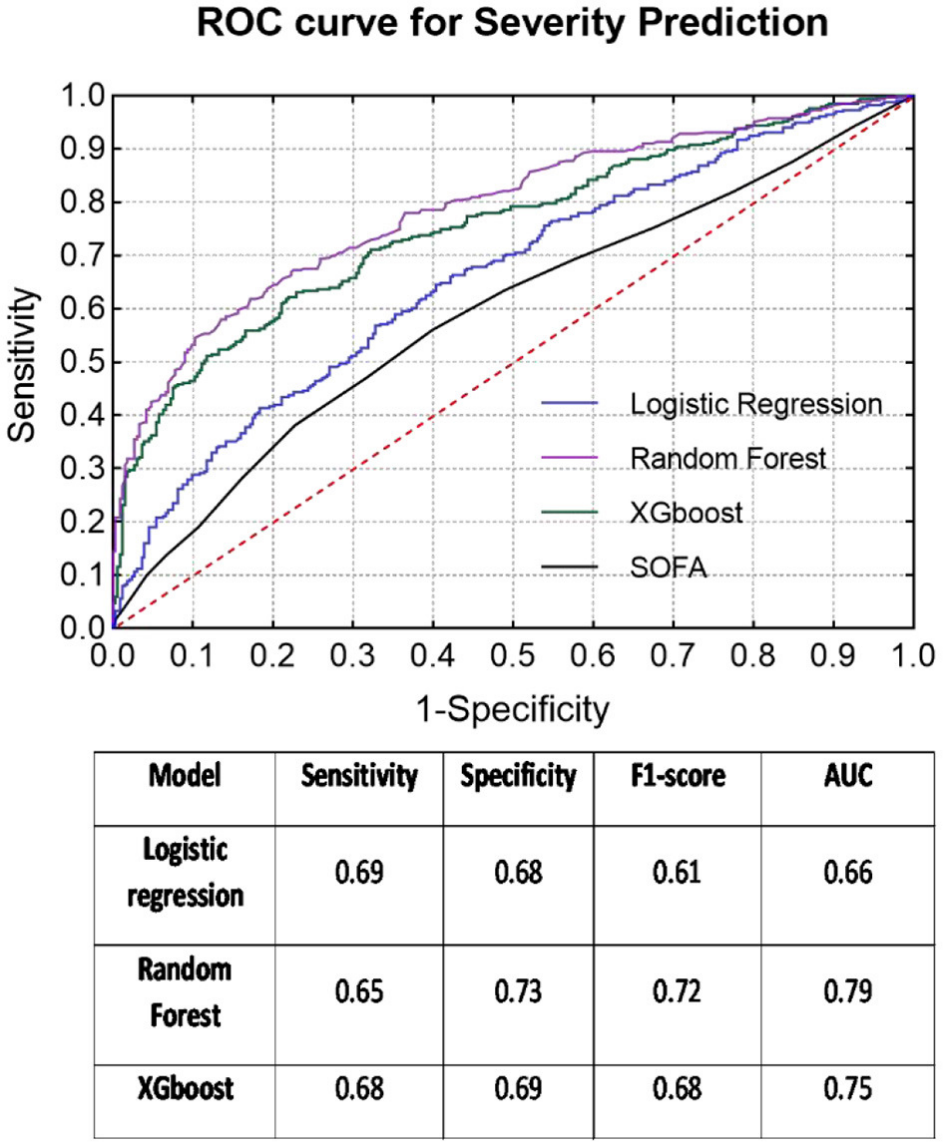
ROC curves of each classifier for severity prediction and classification results in the table below.

The dataset consisted of 1,104 patients with sepsis and 1,120 patients with septic shock. The subgroup with high severity (age: 57.48 ± 16.52 years) was significantly younger than the other subgroup (age: 60.47 ± 16.60 years). As seen in Table 1B, 10 variables related to respiratory [P(v-a)CO_2_/C(a-v)O_2_, oxygenation index, oxygen concentration, SpO_2_], renal (creatinine, adrenaline dosage) and coagulation (invasive blood pressure, central venous pressure, total bilirubin) systems showed no significant differences between the two classes. Among all classifiers, the RF classifier provided the best prediction results for severity (sensitivity = 0.65, specificity = 0.73, F1 score = 0.72, AUC = 0.79) and presented enhanced results compared to the baseline SOFA score (AUC = 0.59) (see Figure 2).

### LOS Prediction

**Figure 3:**
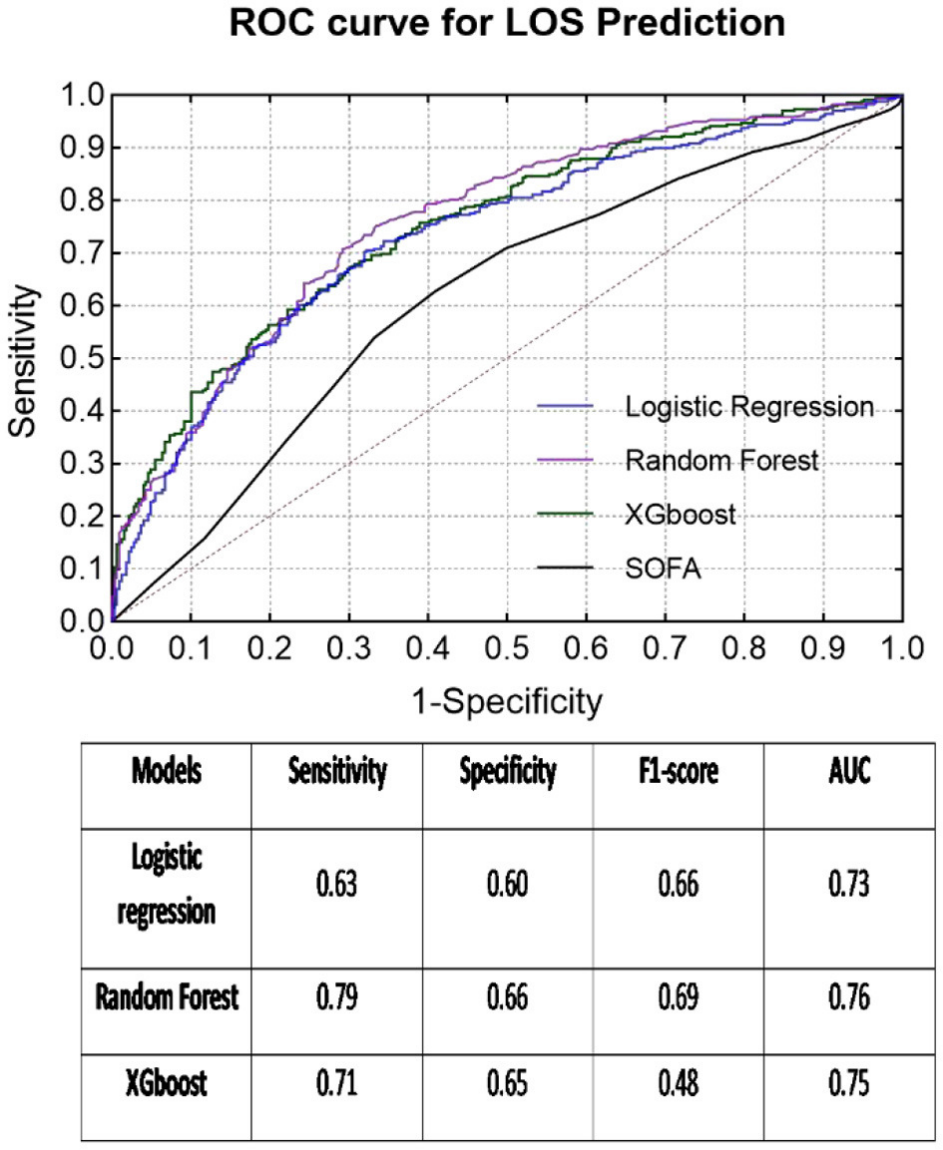
ROC curves of each classifier for LOS prediction and classification results in the table below.

The second quartile (6 days) of all LOS data almost equally divided the group into two classes (≤ 6 days: 1,127 cases, >6 days: 1,097 cases). The patients with longer ICU stays (>6 days) were older (59.82 ± 16.59 years) than the other patients (age: 57.89 ± 16.61 years). Similar to the previous mortality classes, only five variables [perfusion index, P(v-a)CO_2_/C(a-v)O_2_, lactic acid, platelets, and total bilirubin] indicated no significant differences between the LOS subgroups. Meanwhile, the RF model again exhibited the best prediction results for LOS (sensitivity = 0.79, specificity = 0.66, F1 score = 0.69, AUC = 0.76), which was much better than the SOFA score (AUC = 0.62) (see Figure 3).

## Discussion

Our study found that this machine learning method using data within the first 6 h of ICU admission can predict sepsis patients' prognosis, the severity of sepsis (i.e., whether there is septic shock), and the length of stay in the ICU (i.e., whether it was longer than 6 days). Furthermore, the RF classifier had stronger diagnostic power for the three predictions, with areas under the ROC curve of 0.74, 0.79, and 0.76, respectively. After the validation set was verified, its effect was significantly better than that of the traditional SOFA score. This implies that the use of RF predictions in the early stages of ICU admission will enable us to know the possibility of ICU patient outcomes earlier, appropriately allocate medical resources, and optimize treatment behavior.

At present, the diagnosis of sepsis is more specific and clearer with the definition of sepsis 3.0 than the previous two versions. More emphasis should be placed on how we can more accurately predict ICU outcomes after the diagnosis of sepsis (16). As mentioned above, the current treatments for sepsis are still not ideal. Early recognition and correct treatment are closely related to improving prognosis (17). Since sepsis is essentially an out-of-control regulation of the systemic immune response, it is not caused by a single factor. The pathophysiological process is complicated, which leads to large differences in clinical manifestations and disease processes across patients (18). A single diagnostic index is obviously difficult to perform. The sepsis scoring system represented by the SOFA score is used in the diagnosis and treatment of sepsis, which is constantly strengthened by an increasing amount of evidence (19). However, it is difficult to balance the massive data and the complexity of the disease in the ICU treatment of sepsis. With the emergence of large electronic databases and the development of advanced algorithms such as machine learning and data mining, new scoring systems will continue to emerge. Our study identified a relatively good machine learning result, suggesting that the RF method can better predict the 28-day prognosis of patients in the first 6 h after ICU admission. Overall, accurate prediction of the prognosis of ICU patients with sepsis is of great clinical significance. It depends on an appropriate prognostic scoring system. However, how to define and select the “appropriate” scoring system requires the comprehensive judgment of multiple studies and multiple evaluation indicators. In the future, with continuous input of multimodal parameters, more machine learning methods are needed to aggregate data and information from all parties and obtain more accurate conclusions to guide clinical practice.

Compared with the previous two versions of the sepsis guidelines, the largest change was the definition of septic shock (3). At present, septic shock is defined as an inability to maintain blood pressure and the need for vasoactive drugs to maintain circulation after sufficient fluid resuscitation; at this time, lactic acid is >2 mmol/L. For this definition, it may be more necessary to understand the patient's situation and have information from multiple dimensions such as whether this patient is sepsis, what the SOFA score is, whether the patient has undergone fluid resuscitation, what the blood pressure is, whether blood pressure medications are currently being used, and what the lactic acid level is. This makes it even more necessary to use computers as an aid to identify and provide an early alert to ICU staff about this severe sepsis situation. This confirmed that the use of clinical information to define septic shock outperformed models developed based on only administrative data (20). Kim et al. (21) demonstrated that ML classifiers significantly outperformed clinical scores in screening septic shock at ED triage. Combined with machine learning methods, we can see that the RF method can accurately predict patients with septic shock for the first time and determine which patients are more severe. This is of great significance for clinical treatment. Another study also supported our conclusion using a RF classifier to predict sepsis and septic shock (13). In addition, we can also predict which sepsis patients needed longer ICU support through the RF method, and the limited ICU resources can be configured and more efficiently better used. Staziaki et al. (22) reported that SVM and ANN models combining CT findings and clinical parameters improved the prediction of length of stay and ICU admission in torso trauma. Castineira et al. (23) added continuous vital sign information to static clinical data to improve the prediction of length of stay after intubation. Even ELM has been used to determine whether the patient can be discharged within 10 days (24). The use of machine learning algorithms is of great significance to patients with sepsis, and it is better than the traditional SOFA score, which is relatively monotonous in the systematic assessment of organ damage.

The algorithms also played an important role in this study. Before inputting data into the model, imputation of the missing data was necessary. In the future, other imputation methods, such as stochastic regression and tree-based models, can be assessed to compete with the only method, “KNN imputation,” used in this study. The oversampling method “SMOTE” successfully solved the problem of imbalanced datasets, which often leads to a highly biased prediction result, as the model will place more weight on the majority class. In the meantime, some other methods of oversampling can also be tested to improve the classification results. Certainly, as the core of the prediction problem, choosing the best machine learning model is the most important aspect. Therefore, some additional models from the deep learning field, such as artificial neural networks (ANNs) and convolutional neural networks (CNNs), may be applied in future investigations.

There were also some limitations. Firstly, the research subjects came from a single ICU, and there may be bias caused by regional factors. Whether the research conclusions can be extended to other regions needs further research and testing. Secondly, it is necessary to verify that the next step is to implement forward-looking research based on the current research results to further verify the validity and scalability of the model constructed in this study and provide further improvements. In our study, only three subjects have breath rate below 5 bpm, which is only 0.1% of the whole population. It will not lead to high risk of biased dataset according to the inclusion criteria of breath rate > 0 bpm. In the clinical decision-making, the general cut-off point of LOS is 4–5 days while 6 days was chosen here based on the distribution of LOS.

## Conclusion

Machine learning models using the first 6 h of medical data can decently predict mortality, severity, and LOS in the ICU. The overall results demonstrated that the RF model was the best model of classification for all three prediction problems (AUC for all RF models > 0.70) compared to logistic regression and XGBoost models. The prospects of applying machine learning in the ICU are broad, but BCT research is still needed to study the stability of the model and clarify the potential limitations.

## Data Availability Statement

The raw data supporting the conclusions of this article will be made available by the authors, without undue reservation.

## Ethics Statement

Ethical review and approval was not required for the study on human participants in accordance with the local legislation and institutional requirements. The patients/participants provided their written informed consent to participate in this study.

## Author Contributions

YL, WZ, and NH take responsibility for the integrity of the work as a whole, from the inception to the published article. LS, ZX, and FC are responsible for study design and conception. YM, SL, HJ, HW, DL, HC, and XZ are responsible for the data management and statistical analysis. LS drafted the manuscript. All authors revised the manuscript for important intellectual content.

## Conflict of Interest

ZX, FC, YM, and NH were employed by the DHC Software Co. Ltd. The remaining authors declare that the research was conducted in the absence of any commercial or financial relationships that could be construed as a potential conflict of interest.
